# A rare case of primary splenic lymphoma

**DOI:** 10.11604/pamj.2021.39.204.30360

**Published:** 2021-07-16

**Authors:** Issam Loukil, Amine Zouari

**Affiliations:** 1 Service de Chirurgie Générale Tataouine, Tataouine, Tunisie,; 2 Service de Chirurgie Générale Sfax, Sfax, Tunisie

**Keywords:** spleen, lymphoma, primitive

## Image in medicine

A 64-year-old man, with no medical history, presented with an inflammatory syndrome without bacteriological or serological anomaly. Abdominal ultrasound finds a mediosplenic mass of 5cm, hypoechoic heterogeneous without Doppler signals. Computed tomography (CT) scan describes hypodense mass slightly enhanced at the periphery, measuring 57x60x65mm and deforming the splenic hilum, without splenomegaly nor lymphnode (A). Magnetic resonance imaging found a polylobed lesion in T1-T2 isosignal, diffusion hypersignal, crossed by fibrous spans and delimited by a thin wall in T2 hypointense taking the contrast (B, C). The fine needle biopsy was not performed and the treatment decision was to perform a splenectomy. Macroscopic examination described intra splenic tumor polycyclic whitish, crossed by fibrous septa in accordance with imaging (D). Anatomic-pathologic section concluded to a large B cell primary splenic lymphoma. The one-year CT scan did not show any recurrence.

**Figure 1:**
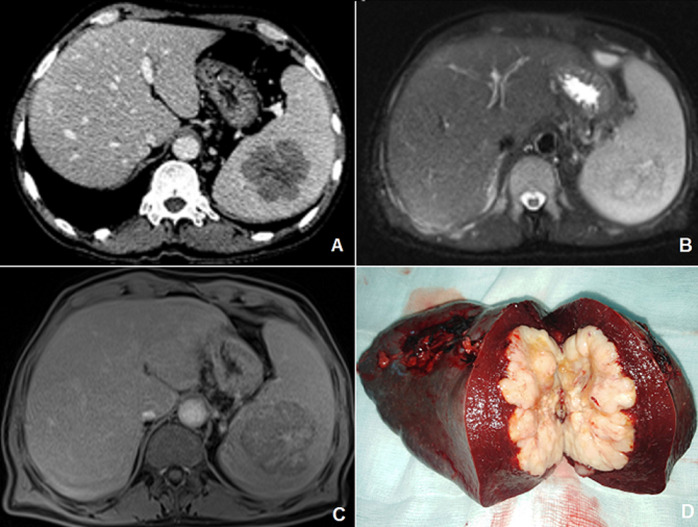
A) CT scan injected during portal phase: hypodense mass slightly enhanced at the periphery, measuring 57x60x65mm and deforming the splenic hilum, without splenomegaly nor lymph node; B, C) magnetic resonance imaging found a polylobed lesion in T1-T2 isosignal, diffusion hypersignal, crossed by fibrous spans and delimited by a thin wall in T2 hypointense taking the contrast; D) intra-splenic tumor mass with a whitish macroscopic appearance, with polycyclic contours and crossed by fibrous partitions

